# (*meso*-5,7,7,12,14,14-Hexamethyl-1,4,8,11-tetra­azacyclo­tetra­deca-4,11-diene)nickel(II) bis­[*O*,*O*′-bis(4-methyl­phen­yl) dithio­phosphate]

**DOI:** 10.1107/S1600536809030955

**Published:** 2009-08-08

**Authors:** Bin Xie, Yang-Guang Xiang, Li-Ke Zou, Xiu-Li Chang, Chang-You Ji

**Affiliations:** a College of Chemistry and Pharmaceutical Engineering, Sichuan University of Science and Engineering, 643000 Zigong, Sichuan, People’s Republic of China; b College of Environment and Chemical Engineering, Xi’an Polytechnic University, 710048 Xi’an, Shanxi, People’s Republic of China

## Abstract

In the title compound, [Ni(C_16_H_32_N_4_)](C_14_H_14_O_2_PS_2_)_2_ or [Ni(*trans*[14]dien)][S_2_P(OC_6_H_4_Me-4)_2_]_2_, where *trans*[14]dien is *meso*-5,7,7,12,14,14-hexa­methyl-1,4,8,11-tetra­azacyclo­tetra­deca-4,11-diene, the Ni^II^ ion lies across a centre of inversion and is four-coordinated in a relatively undistorted square-planar arrangement by the four N atoms of the macrocyclic ligand *trans*[14]dien. The two *O*,*O*′-di(4-methyl­phen­yl)dithio­phos­phates act as counter-ions to balance the charge. Important geometric data include Ni—N = 1.9135 (16) and 1.9364 (15) Å.

## Related literature

For related structures, see: Xie *et al.* (2008 ▶). For bond-length data, see: Allen *et al.* (1987 ▶). 
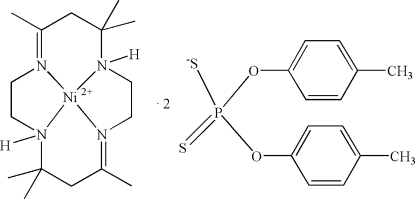


## Experimental

### 

#### Crystal data

[Ni(C_16_H_32_N_4_)](C_14_H_14_O_2_PS_2_)_2_
*M* *_r_* = 957.85
Triclinic, 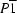

*a* = 8.0044 (6) Å
*b* = 10.0996 (8) Å
*c* = 16.4004 (12) Å
α = 80.418 (1)°
β = 81.333 (1)°
γ = 69.836 (1)°
*V* = 1220.95 (16) Å^3^
*Z* = 1
Mo *K*α radiation
μ = 0.68 mm^−1^
*T* = 278 K
0.18 × 0.14 × 0.10 mm

#### Data collection

Bruker SMART CCD area-detector diffractometer
Absorption correction: multi-scan (*SADABS*; Bruker, 2001 ▶) *T*_min_ = 0.888, *T*_max_ = 0.935
6525 measured reflections
4305 independent reflections
3756 reflections with *I* > 2σ(*I*)
*R*_int_ = 0.012

#### Refinement

*R*[*F* ^2^ > 2σ(*F* ^2^)] = 0.032
*wR*(*F* ^2^) = 0.085
*S* = 1.04
4305 reflections
273 parameters
H atoms treated by a mixture of independent and constrained refinement
Δρ_max_ = 0.50 e Å^−3^
Δρ_min_ = −0.34 e Å^−3^

### 

Data collection: *SMART* (Bruker, 2001 ▶); cell refinement: *SAINT* (Bruker, 2001 ▶); data reduction: *SAINT*; program(s) used to solve structure: *SHELXTL* (Sheldrick, 2008 ▶); program(s) used to refine structure: *SHELXTL*; molecular graphics: *ORTEP-3* (Farrugia, 1997 ▶); software used to prepare material for publication: *SHELXTL*.

## Supplementary Material

Crystal structure: contains datablocks I, global. DOI: 10.1107/S1600536809030955/dn2479sup1.cif

Structure factors: contains datablocks I. DOI: 10.1107/S1600536809030955/dn2479Isup2.hkl

Additional supplementary materials:  crystallographic information; 3D view; checkCIF report

## Figures and Tables

**Fig. 1.**
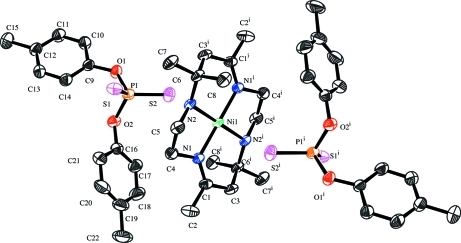
The molecular structure of compound, showing the atom-numbering scheme. Displacement ellipsoids are drawn at the 30% probability level.H atoms have been omitted for the sake of clarity.[Symmetry code: (i) -x + 1, -y + 1, -z + 1].

## supplementary crystallographic information

### Comment

As part of an investigation to the potential applications of tetramine macrocyclic transition metal complexs as artificial enzyme models, we have recently reported the structures of [Ni(teta)][S_2_P(OCH_2_Ph)_2_]_2_, where teta is *meso*-5,5,7,12,12,14- hexamethyl-1,4,8,11-tetraazacyclotetradecane (Xie *et al.*, 2008). Here we report the structure of [Ni(*trans*[14]dien)] [S_2_P(OC_6_H_4_Me-4)_2_]_2_, where *trans*[14]dien is *meso*-5,7,7,12,14,14- hexamethyl-1,4,8,11-tetraazacyclotetradeca-4,11-diene.

In the title compound, the Ni^II^ atom exhibits a relatively undistorted square-planar geometry (Fig.1), which lies on an inversion centre and is coordinated by four N atoms of the macrocyclic ligand *trans*[14]dien. The two *O*,*O*'-di(4-methylphenyl) dithiophosphates only act as counter-ions to balance the charge.All the bond lengths and angles in the complex are generally within normal ranges (Allen *et al.*, 1987).

### Experimental

*meso*-5,7,7,12,14,14-Hexamethyl-1,4,8,1 l-tetraazacyclotetradeca-4, 11-diene nickel(II) perchlorate(2 mmol 1.28 g) was added to a solution of diethylammonium *O*,*O*'-di(4-methylphenyl)dithiophosphate (4 mmol 1.534 g) in 60 ml methanol. The mixture was refluxed for 8 h at 353 K and then filtered. The filtrate was kept at room temperature and orange block crystals were obtained after 4 days.

### Refinement

H atoms on C were fixed geometrically and treated as riding, with C—H = 0.97 Å (methylene), 0.96Å (methyl) or 0.93Å (aromatic) and *U*_iso_(H) = 1.2*U*_eq_(*C*,methylene and aromatic) or *U*_iso_(H) = 1.5*U*_eq_(*C*,methyl). The H atoms on N were determined with difference Fourier syntheses and refined isotropically.

### Crystal data

**Table:**

[Ni(C_16_H_32_N_4_)](C_14_H_14_O_2_PS_2_)_2_	*Z* = 1
*M**_r_* = 957.85	*F*(000) = 506
Triclinic, *P*1	*D*_x_ = 1.303 Mg m^−^^3^
Hall symbol: -P 1	Mo *K*α radiation, λ = 0.71073 Å
*a* = 8.0044 (6) Å	Cell parameters from 3628 reflections
*b* = 10.0996 (8) Å	θ = 2.4–28.3°
*c* = 16.4004 (12) Å	µ = 0.68 mm^−^^1^
α = 80.418 (1)°	*T* = 278 K
β = 81.333 (1)°	Block, orange
γ = 69.836 (1)°	0.18 × 0.14 × 0.10 mm
*V* = 1220.95 (16) Å^3^	

### Data collection

**Table:**

Bruker SMART CCD area-detector diffractometer	4305 independent reflections
Radiation source: fine-focus sealed tube	3756 reflections with *I* > 2σ(*I*)
graphite	*R*_int_ = 0.012
φ and ω scans	θ_max_ = 25.1°, θ_min_ = 2.2°
Absorption correction: multi-scan (*SADABS*; Bruker, 2001)	*h* = −9→6
*T*_min_ = 0.888, *T*_max_ = 0.935	*k* = −12→11
6525 measured reflections	*l* = −18→19

### Refinement

**Table:**

Refinement on *F*^2^	Secondary atom site location: difference Fourier map
Least-squares matrix: full	Hydrogen site location: inferred from neighbouring sites
*R*[*F*^2^ > 2σ(*F*^2^)] = 0.032	H atoms treated by a mixture of independent and constrained refinement
*wR*(*F*^2^) = 0.085	*w* = 1/[σ^2^(*F*_o_^2^) + (0.0404*P*)^2^ + 0.4007*P*] where *P* = (*F*_o_^2^ + 2*F*_c_^2^)/3
*S* = 1.04	(Δ/σ)_max_ < 0.001
4305 reflections	Δρ_max_ = 0.50 e Å^−^^3^
273 parameters	Δρ_min_ = −0.33 e Å^−^^3^
0 restraints	Extinction correction: *SHELXL97*, Fc^*^=kFc[1+0.001xFc^2^λ^3^/sin(2θ)]^-1/4^
Primary atom site location: structure-invariant direct methods	Extinction coefficient: 0.0094 (11)

### Special details

**Table:**

Geometry. All e.s.d.'s (except the e.s.d. in the dihedral angle between two l.s. planes) are estimated using the full covariance matrix. The cell e.s.d.'s are taken into account individually in the estimation of e.s.d.'s in distances, angles and torsion angles; correlations between e.s.d.'s in cell parameters are only used when they are defined by crystal symmetry. An approximate (isotropic) treatment of cell e.s.d.'s is used for estimating e.s.d.'s involving l.s. planes.
Refinement. Refinement of *F*^2^ against ALL reflections. The weighted *R*-factor *wR* and goodness of fit *S* are based on *F*^2^, conventional *R*-factors *R* are based on *F*, with *F* set to zero for negative *F*^2^. The threshold expression of *F*^2^ > σ(*F*^2^) is used only for calculating *R*-factors(gt) *etc*. and is not relevant to the choice of reflections for refinement. *R*-factors based on *F*^2^ are statistically about twice as large as those based on *F*, and *R*- factors based on ALL data will be even larger.

### Fractional atomic coordinates and isotropic or equivalent isotropic displacement parameters (Å^2^)

**Table:**

	*x*	*y*	*z*	*U*_iso_*/*U*_eq_	
Ni1	0.5000	0.5000	0.5000	0.03886 (12)	
S1	0.51679 (8)	0.93375 (7)	0.29986 (4)	0.06565 (19)	
S2	0.80706 (8)	0.63426 (7)	0.38281 (4)	0.06475 (18)	
P1	0.74959 (7)	0.78569 (6)	0.28970 (4)	0.05003 (16)	
O1	0.9135 (2)	0.84827 (17)	0.27241 (10)	0.0615 (4)	
O2	0.7837 (2)	0.72019 (16)	0.20251 (9)	0.0582 (4)	
N1	0.3850 (2)	0.48109 (17)	0.41047 (10)	0.0448 (4)	
N2	0.3765 (2)	0.70211 (16)	0.47728 (11)	0.0437 (4)	
C1	0.3764 (3)	0.3675 (2)	0.38841 (13)	0.0507 (5)	
C2	0.2623 (4)	0.3675 (3)	0.32375 (19)	0.0806 (8)	
H2A	0.1387	0.4131	0.3418	0.121*	
H2B	0.2803	0.2713	0.3157	0.121*	
H2C	0.2948	0.4180	0.2723	0.121*	
C3	0.4795 (3)	0.2249 (2)	0.42779 (15)	0.0581 (6)	
H3A	0.4977	0.1572	0.3891	0.070*	
H3B	0.4060	0.1994	0.4763	0.070*	
C4	0.2860 (3)	0.6213 (2)	0.36823 (15)	0.0578 (6)	
H4A	0.1864	0.6155	0.3436	0.069*	
H4B	0.3640	0.6542	0.3247	0.069*	
C5	0.2199 (3)	0.7204 (2)	0.43308 (16)	0.0586 (6)	
H5A	0.1723	0.8178	0.4076	0.070*	
H5B	0.1264	0.6972	0.4714	0.070*	
C6	0.3396 (3)	0.7936 (2)	0.54526 (14)	0.0509 (5)	
C7	0.2575 (4)	0.9514 (2)	0.51212 (18)	0.0716 (7)	
H7A	0.3355	0.9768	0.4665	0.107*	
H7B	0.2426	1.0087	0.5556	0.107*	
H7C	0.1432	0.9671	0.4936	0.107*	
C8	0.2158 (3)	0.7501 (2)	0.61610 (16)	0.0668 (6)	
H8A	0.0998	0.7719	0.5978	0.100*	
H8B	0.2055	0.8010	0.6621	0.100*	
H8C	0.2639	0.6498	0.6332	0.100*	
C9	0.9401 (3)	0.9521 (2)	0.20917 (14)	0.0520 (5)	
C10	1.0778 (3)	0.9987 (3)	0.21639 (16)	0.0643 (6)	
H10	1.1433	0.9634	0.2621	0.077*	
C11	1.1198 (4)	1.0978 (3)	0.15607 (17)	0.0743 (7)	
H11	1.2137	1.1288	0.1618	0.089*	
C12	1.0265 (4)	1.1519 (3)	0.08774 (16)	0.0706 (7)	
C13	0.8887 (4)	1.1040 (3)	0.08206 (17)	0.0816 (8)	
H13	0.8233	1.1392	0.0363	0.098*	
C14	0.8432 (4)	1.0046 (3)	0.14216 (17)	0.0733 (7)	
H14	0.7485	0.9742	0.1370	0.088*	
C15	1.0710 (5)	1.2625 (4)	0.0212 (2)	0.1070 (11)	
H15A	1.0213	1.2641	−0.0288	0.161*	
H15B	1.1985	1.2387	0.0103	0.161*	
H15C	1.0213	1.3544	0.0402	0.161*	
C16	0.6734 (3)	0.6529 (2)	0.18118 (13)	0.0562 (5)	
C17	0.7294 (4)	0.5084 (3)	0.18933 (17)	0.0736 (7)	
H17	0.8360	0.4553	0.2118	0.088*	
C18	0.6240 (6)	0.4413 (4)	0.1634 (2)	0.0970 (10)	
H18	0.6617	0.3426	0.1690	0.116*	
C19	0.4665 (6)	0.5173 (5)	0.13009 (19)	0.0973 (11)	
C20	0.4129 (5)	0.6620 (4)	0.12418 (18)	0.0931 (9)	
H20	0.3046	0.7151	0.1032	0.112*	
C21	0.5151 (4)	0.7320 (3)	0.14848 (16)	0.0724 (7)	
H21	0.4774	0.8308	0.1428	0.087*	
C22	0.3564 (7)	0.4413 (5)	0.1003 (2)	0.152 (2)	
H22A	0.2336	0.4791	0.1223	0.227*	
H22B	0.4016	0.3415	0.1192	0.227*	
H22C	0.3644	0.4555	0.0407	0.227*	
H1	0.454 (2)	0.725 (2)	0.4405 (9)	0.050 (6)*	

### Atomic displacement parameters (Å^2^)

**Table:**

	*U*^11^	*U*^22^	*U*^33^	*U*^12^	*U*^13^	*U*^23^
Ni1	0.03462 (19)	0.03290 (18)	0.0518 (2)	−0.01460 (14)	−0.00187 (14)	−0.00726 (14)
S1	0.0497 (3)	0.0674 (4)	0.0815 (4)	−0.0103 (3)	−0.0107 (3)	−0.0296 (3)
S2	0.0511 (3)	0.0700 (4)	0.0695 (4)	−0.0252 (3)	0.0033 (3)	0.0047 (3)
P1	0.0398 (3)	0.0550 (3)	0.0595 (3)	−0.0211 (2)	−0.0010 (2)	−0.0099 (3)
O1	0.0508 (9)	0.0682 (10)	0.0724 (10)	−0.0338 (8)	−0.0172 (7)	0.0134 (8)
O2	0.0550 (9)	0.0629 (9)	0.0610 (9)	−0.0280 (7)	0.0116 (7)	−0.0162 (7)
N1	0.0388 (8)	0.0465 (9)	0.0529 (9)	−0.0193 (7)	−0.0021 (7)	−0.0079 (7)
N2	0.0369 (8)	0.0369 (8)	0.0579 (10)	−0.0161 (7)	0.0019 (7)	−0.0051 (7)
C1	0.0440 (11)	0.0584 (12)	0.0579 (12)	−0.0248 (10)	0.0044 (9)	−0.0209 (10)
C2	0.0703 (17)	0.094 (2)	0.093 (2)	−0.0299 (15)	−0.0153 (15)	−0.0417 (16)
C3	0.0655 (14)	0.0489 (12)	0.0706 (14)	−0.0302 (11)	0.0049 (11)	−0.0222 (10)
C4	0.0523 (12)	0.0564 (13)	0.0676 (14)	−0.0197 (10)	−0.0186 (11)	−0.0003 (11)
C5	0.0453 (12)	0.0472 (12)	0.0810 (16)	−0.0100 (9)	−0.0140 (11)	−0.0058 (11)
C6	0.0502 (12)	0.0365 (10)	0.0670 (13)	−0.0176 (9)	0.0088 (10)	−0.0150 (9)
C7	0.0739 (16)	0.0372 (11)	0.0992 (19)	−0.0154 (11)	0.0037 (14)	−0.0134 (12)
C8	0.0630 (15)	0.0575 (14)	0.0740 (16)	−0.0199 (11)	0.0196 (12)	−0.0154 (12)
C9	0.0511 (12)	0.0509 (12)	0.0578 (13)	−0.0229 (10)	−0.0060 (10)	−0.0034 (10)
C10	0.0540 (13)	0.0792 (16)	0.0664 (14)	−0.0344 (12)	−0.0110 (11)	0.0047 (12)
C11	0.0684 (16)	0.0855 (18)	0.0819 (18)	−0.0482 (14)	−0.0039 (14)	0.0006 (14)
C12	0.0870 (18)	0.0644 (15)	0.0668 (16)	−0.0392 (14)	0.0014 (14)	−0.0026 (12)
C13	0.108 (2)	0.0776 (18)	0.0716 (17)	−0.0462 (17)	−0.0337 (16)	0.0127 (14)
C14	0.0813 (17)	0.0724 (16)	0.0822 (18)	−0.0450 (14)	−0.0304 (14)	0.0095 (14)
C15	0.144 (3)	0.106 (2)	0.087 (2)	−0.076 (2)	−0.005 (2)	0.0168 (18)
C16	0.0642 (14)	0.0647 (14)	0.0456 (11)	−0.0305 (12)	0.0101 (10)	−0.0167 (10)
C17	0.0858 (18)	0.0643 (15)	0.0735 (17)	−0.0294 (14)	0.0077 (14)	−0.0201 (13)
C18	0.142 (3)	0.084 (2)	0.085 (2)	−0.062 (2)	0.014 (2)	−0.0307 (17)
C19	0.133 (3)	0.134 (3)	0.0614 (17)	−0.086 (3)	0.0001 (18)	−0.0301 (18)
C20	0.100 (2)	0.132 (3)	0.0655 (17)	−0.053 (2)	−0.0194 (16)	−0.0189 (18)
C21	0.0818 (18)	0.0788 (17)	0.0612 (15)	−0.0287 (14)	−0.0103 (13)	−0.0133 (13)
C22	0.223 (5)	0.224 (5)	0.090 (2)	−0.169 (5)	−0.011 (3)	−0.040 (3)

### Geometric parameters (Å, °)

**Table:**

Ni1—N1	1.9135 (16)	C7—H7C	0.9600
Ni1—N1^i^	1.9135 (16)	C8—H8A	0.9600
Ni1—N2^i^	1.9364 (15)	C8—H8B	0.9600
Ni1—N2	1.9364 (15)	C8—H8C	0.9600
S1—P1	1.9505 (8)	C9—C10	1.366 (3)
S2—P1	1.9575 (8)	C9—C14	1.367 (3)
P1—O1	1.6131 (14)	C10—C11	1.377 (3)
P1—O2	1.6216 (16)	C10—H10	0.9300
O1—C9	1.395 (3)	C11—C12	1.371 (4)
O2—C16	1.396 (3)	C11—H11	0.9300
N1—C1	1.285 (2)	C12—C13	1.369 (4)
N1—C4	1.476 (3)	C12—C15	1.522 (4)
N2—C5	1.483 (3)	C13—C14	1.388 (3)
N2—C6	1.497 (3)	C13—H13	0.9300
C1—C3	1.484 (3)	C14—H14	0.9300
C1—C2	1.500 (3)	C15—H15A	0.9600
C2—H2A	0.9600	C15—H15B	0.9600
C2—H2B	0.9600	C15—H15C	0.9600
C2—H2C	0.9600	C16—C17	1.360 (3)
C3—C6^i^	1.518 (3)	C16—C21	1.377 (4)
C3—H3A	0.9700	C17—C18	1.396 (4)
C3—H3B	0.9700	C17—H17	0.9300
C4—C5	1.494 (3)	C18—C19	1.371 (5)
C4—H4A	0.9700	C18—H18	0.9300
C4—H4B	0.9700	C19—C20	1.365 (5)
C5—H5A	0.9700	C19—C22	1.525 (4)
C5—H5B	0.9700	C20—C21	1.385 (4)
C6—C3^i^	1.518 (3)	C20—H20	0.9300
C6—C8	1.519 (3)	C21—H21	0.9300
C6—C7	1.537 (3)	C22—H22A	0.9600
C7—H7A	0.9600	C22—H22B	0.9600
C7—H7B	0.9600	C22—H22C	0.9600
			
N1—Ni1—N1^i^	180.000 (1)	H7A—C7—H7B	109.5
N1—Ni1—N2^i^	94.15 (7)	C6—C7—H7C	109.5
N1^i^—Ni1—N2^i^	85.85 (7)	H7A—C7—H7C	109.5
N1—Ni1—N2	85.85 (7)	H7B—C7—H7C	109.5
N1^i^—Ni1—N2	94.15 (7)	C6—C8—H8A	109.5
N2^i^—Ni1—N2	180.000 (1)	C6—C8—H8B	109.5
O1—P1—O2	96.75 (9)	H8A—C8—H8B	109.5
O1—P1—S1	112.77 (7)	C6—C8—H8C	109.5
O2—P1—S1	111.07 (7)	H8A—C8—H8C	109.5
O1—P1—S2	106.04 (6)	H8B—C8—H8C	109.5
O2—P1—S2	110.96 (7)	C10—C9—C14	120.0 (2)
S1—P1—S2	117.27 (4)	C10—C9—O1	115.11 (19)
C9—O1—P1	127.94 (14)	C14—C9—O1	124.9 (2)
C16—O2—P1	122.66 (13)	C9—C10—C11	120.1 (2)
C1—N1—C4	119.78 (18)	C9—C10—H10	120.0
C1—N1—Ni1	128.75 (15)	C11—C10—H10	120.0
C4—N1—Ni1	111.31 (12)	C12—C11—C10	121.6 (2)
C5—N2—C6	115.10 (16)	C12—C11—H11	119.2
C5—N2—Ni1	107.47 (12)	C10—C11—H11	119.2
C6—N2—Ni1	119.17 (13)	C13—C12—C11	117.2 (2)
C5—N2—H1	106.3 (14)	C13—C12—C15	120.9 (3)
C6—N2—H1	107.6 (14)	C11—C12—C15	121.9 (3)
Ni1—N2—H1	99.3 (14)	C12—C13—C14	122.3 (2)
N1—C1—C3	120.94 (19)	C12—C13—H13	118.8
N1—C1—C2	123.7 (2)	C14—C13—H13	118.8
C3—C1—C2	115.32 (19)	C9—C14—C13	118.8 (2)
C1—C2—H2A	109.5	C9—C14—H14	120.6
C1—C2—H2B	109.5	C13—C14—H14	120.6
H2A—C2—H2B	109.5	C12—C15—H15A	109.5
C1—C2—H2C	109.5	C12—C15—H15B	109.5
H2A—C2—H2C	109.5	H15A—C15—H15B	109.5
H2B—C2—H2C	109.5	C12—C15—H15C	109.5
C1—C3—C6^i^	117.74 (17)	H15A—C15—H15C	109.5
C1—C3—H3A	107.9	H15B—C15—H15C	109.5
C6^i^—C3—H3A	107.9	C17—C16—C21	120.8 (2)
C1—C3—H3B	107.9	C17—C16—O2	118.9 (2)
C6^i^—C3—H3B	107.9	C21—C16—O2	120.3 (2)
H3A—C3—H3B	107.2	C16—C17—C18	118.9 (3)
N1—C4—C5	106.65 (18)	C16—C17—H17	120.6
N1—C4—H4A	110.4	C18—C17—H17	120.6
C5—C4—H4A	110.4	C19—C18—C17	121.7 (3)
N1—C4—H4B	110.4	C19—C18—H18	119.2
C5—C4—H4B	110.4	C17—C18—H18	119.2
H4A—C4—H4B	108.6	C20—C19—C18	117.9 (3)
N2—C5—C4	106.26 (17)	C20—C19—C22	121.5 (4)
N2—C5—H5A	110.5	C18—C19—C22	120.6 (4)
C4—C5—H5A	110.5	C19—C20—C21	121.9 (3)
N2—C5—H5B	110.5	C19—C20—H20	119.1
C4—C5—H5B	110.5	C21—C20—H20	119.1
H5A—C5—H5B	108.7	C16—C21—C20	118.9 (3)
N2—C6—C3^i^	106.09 (16)	C16—C21—H21	120.6
N2—C6—C8	110.29 (17)	C20—C21—H21	120.6
C3^i^—C6—C8	112.3 (2)	C19—C22—H22A	109.5
N2—C6—C7	110.74 (19)	C19—C22—H22B	109.5
C3^i^—C6—C7	106.99 (18)	H22A—C22—H22B	109.5
C8—C6—C7	110.33 (18)	C19—C22—H22C	109.5
C6—C7—H7A	109.5	H22A—C22—H22C	109.5
C6—C7—H7B	109.5	H22B—C22—H22C	109.5
			
O2—P1—O1—C9	61.8 (2)	C5—N2—C6—C7	−55.6 (2)
S1—P1—O1—C9	−54.5 (2)	Ni1—N2—C6—C7	174.50 (14)
S2—P1—O1—C9	175.93 (17)	P1—O1—C9—C10	171.21 (17)
O1—P1—O2—C16	−178.20 (17)	P1—O1—C9—C14	−10.6 (4)
S1—P1—O2—C16	−60.61 (18)	C14—C9—C10—C11	−0.4 (4)
S2—P1—O2—C16	71.73 (17)	O1—C9—C10—C11	177.9 (2)
N2^i^—Ni1—N1—C1	10.52 (18)	C9—C10—C11—C12	−0.2 (4)
N2—Ni1—N1—C1	−169.48 (18)	C10—C11—C12—C13	0.4 (4)
N2^i^—Ni1—N1—C4	−174.05 (14)	C10—C11—C12—C15	179.4 (3)
N2—Ni1—N1—C4	5.95 (14)	C11—C12—C13—C14	−0.1 (5)
N1^i^—Ni1—N2—C5	−157.85 (14)	C15—C12—C13—C14	−179.2 (3)
N1^i^—Ni1—N2—C6	−24.62 (14)	C10—C9—C14—C13	0.6 (4)
C4—N1—C1—C3	177.86 (19)	O1—C9—C14—C13	−177.5 (2)
Ni1—N1—C1—C3	−7.0 (3)	C12—C13—C14—C9	−0.4 (5)
C4—N1—C1—C2	−3.3 (3)	P1—O2—C16—C17	−101.5 (2)
Ni1—N1—C1—C2	171.82 (17)	P1—O2—C16—C21	81.8 (2)
N1—C1—C3—C6^i^	−33.5 (3)	C21—C16—C17—C18	0.4 (4)
C2—C1—C3—C6^i^	147.6 (2)	O2—C16—C17—C18	−176.3 (2)
C1—N1—C4—C5	143.51 (19)	C16—C17—C18—C19	0.1 (4)
Ni1—N1—C4—C5	−32.4 (2)	C17—C18—C19—C20	−1.2 (5)
C6—N2—C5—C4	179.55 (17)	C17—C18—C19—C22	178.5 (3)
Ni1—N2—C5—C4	−45.1 (2)	C18—C19—C20—C21	1.9 (5)
N1—C4—C5—N2	49.9 (2)	C22—C19—C20—C21	−177.8 (3)
C5—N2—C6—C3^i^	−171.36 (17)	C17—C16—C21—C20	0.2 (4)
Ni1—N2—C6—C3^i^	58.76 (19)	O2—C16—C21—C20	176.9 (2)
C5—N2—C6—C8	66.8 (2)	C19—C20—C21—C16	−1.4 (4)
Ni1—N2—C6—C8	−63.1 (2)		

Symmetry codes: (i) −*x*+1, −*y*+1, −*z*+1.
